# The utility of point of care ultrasonography (POCUS)

**DOI:** 10.1016/j.amsu.2021.102982

**Published:** 2021-11-02

**Authors:** Ahmed Hashim, Muhammad Junaid Tahir, Irfan Ullah, Muhammad Sohaib Asghar, Haziq Siddiqi, Zohaib Yousaf

**Affiliations:** a Faculty of Medicine, Ain Shams University, Cairo, Egypt; b Lahore General Hospital, Lahore, 54000, Pakistan; c Kabir Medical College, Gandhara University, Peshawar, Pakistan; d Dow University of Health Sciences–Ojha Campus, Karachi, Pakistan; e University of California, San Francisco, United States; f Hamad Medical Corporation, Doha, Qatar

**Keywords:** Radiology, Imaging techniques, POCUS, Ultrasound, Medical education, Diagnosis

## 1 Introduction

Point of care ultrasonography (POCUS) is advanced diagnostic ultrasonography that is performed and interpreted by the attending physician as a bedside test [1]. POCUS has been widely used in many disciplines as a rapid diagnostic tool, especially in emergency medicine. POCUS has been used to aid the diagnosis of multiple medical conditions ranging from acute appendicitis, airway compromise, abdominal aortic aneurysm, traumatic injury assessment [2]. The relatively fast use has made it a potential option in situations where a formal radiological investigation may delay the diagnosis. Additionally, the ever-increasing demands of other diagnostic imaging and interventional radiological procedures have underscored the importance of non-radiologists physicians' contribution to radiological diagnosis through POCUS [3].

There are several advantages of incorporating POCUS in daily clinical practice, with the major one being integrating sonographic findings with history and clinical examination at the patient's bedside [4]. In addition, POCUS performed by the primary clinician reduces the need to involve a second clinician and avoids the need for patient transfer to a separate ultrasonography room. POCUS is a cost-effective approach that directly and indirectly saves healthcare expenses at both national and international scales [5].

## 2 The extent of POCUS usage

POCUS use and implementation have expanded significantly over the last decade and a half. However, despite being widely used across different medical specializations, no unifying global estimation of its use exists. There is significant variability in the access of healthcare providers to POCUS across Europe [6]. Mengel-Jorgensen et al. [7] demonstrated varied applications of POCUS, with more than 40% in Greenland and Germany to less than 1% usage in Catalonia, Austria, Sweden, and Denmark health care centers. In France, POCUS availability in the emergency department was as high as 52% in 2011 which has increased to 71% in 2016 [8]. Additionally, in more than 80% of the Danish emergency departments, POCUS has been available to emergency physicians [9]. In rural Canada, general and emergency practitioners’ (EPs), access to POCUS has increased from 60% access in 2013 to more than 90% access in 2019, with 44%–76% practitioners have reported using it [10]. In the USA, ultrasound training is now integrated into EP training [11].

This increase in POCUS availability and use was not limited to Europe and North America. In China, more than half of emergency department physicians have reported having access to POCUS, with 43% reporting using it in their clinical work [12]. Ahn et al. [13] demonstrated that POCUS was available in all surveyed emergency departments of South Korea, with 82.7% of respondents used POCUS daily on adult patients.

## 3 Indications of POCUS (Fig. 1)

### 3.1 Cardiovascular and pulmonary indications

The use of POCUS in clinical practice has been evolving significantly over the past few years. The accuracy of POCUS in diagnosing pulmonary conditions is equivalent or even higher than laboratory markers in diagnosing specific pulmonary conditions [14]. As an example, detection of bilateral pulmonary B lines is more specific (100%) and sensitive (95%) than elevated pro-brain natriuretic peptide (pro-BNP) levels, which have 92% sensitivity, 89% specificity in diagnosing acute decompensated heart failure [15].

**Fig. 1:**
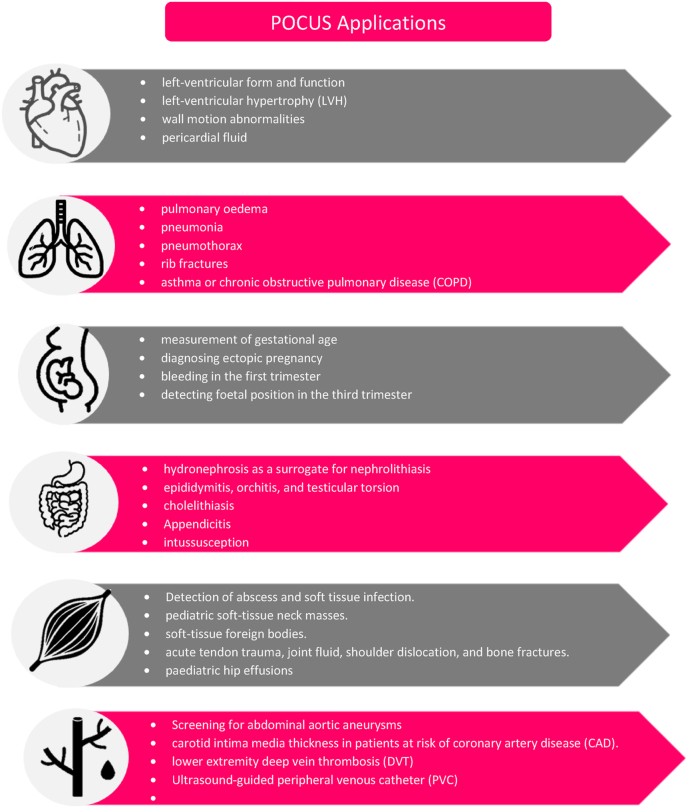
Applications of point of care ultrasound (POCUS) in the cardiovascular, respiratory, obstetrical, gastrointestinal, musculoskeletal, and vascular system.

The use of POCUS by the EPs in determining left ventricular ejection fraction (LVEF) has an excellent overall agreement (84 and 93%) between EPs and cardiology sonographers [16]. POCUS has high sensitivity (96–100%) in the diagnosis of pericardial effusion but with more false-negative findings in small volume effusion [16]. The utility of POCUS in screening abdominal aortic aneurysms in the emergency department has shown 100% accuracy for aneurysms detection if its size was more than 3 cm in diameter [17]. Diagnosing deep venous thrombosis using two-point compression techniques by GPs has 90% sensitivity and 97% specificity [18].

### 3.2 Abdominal and obstetrical indications

Diagnosis of nephrolithiasis in high-risk patients using POCUS showed no difference from computerized tomography (CT) scan and resulted in lower radiation exposure and reduced emergency department length of stay [19]. This trend of reduction in length of stay with POCUS use was also observed in diagnoses of biliary disease [20]. POCUS has an excellent positive predictive value for the diagnosis of appendicitis but has a low negative predictive value [21].

Ultrasonography can also be used in the diagnosis of small bowel obstruction. Abnormal peristalsis, small bowel dilatation, intraperitoneally free fluid, and small bowel wall edema suggest small bowel obstruction [22]. Different studies have demonstrated high sensitivity in the emergency department-based diagnosis of small bowel obstruction using POCUS when compared to radiology-based ultrasound and CT scan [23]. Furthermore, there was 100% agreement between GPs and radiologists in diagnosing ascites [24].

Stein et al. [25], demonstrated a pooled sensitivity approaching 99% and specificity of 42–89% in confirming empty uterus in diagnosing suspicious ectopic pregnancy using POCUS. Evaluation of early pregnancy with POCUS decreases the overall length of hospital stay [26]. Of note, GPs using POCUS have 100% accuracy in diagnosing fetal heart activity, hence excluding or establishing threatened and missed abortions [27]. Both GPs and EPs had 100% accuracy in determining the fetal position in the 3rd trimester of pregnancy [28].

### 3.3 Recommendations

Patient care and patient safety should always be a priority in decision-making. Authors recommend on the job formal training of both GPs and EPs with certifications before independent use of POCUS. This should be followed by widespread integration of POCUS in routine healthcare. This will lead to the delivery of quality healthcare services cost-effectively.

## 4 Conclusion

POCUS applications in medical diagnosis are progressively expanding in almost all medical specialties. Several advantages include quick diagnoses, cost-effectiveness, and shorter hospital stays. The need of the hour is to establish a unified, integrated formal curriculum and adequate training for safe and effective use of POCUS.

## Financial Support

This research did not receive any specific grant from funding agencies in the public, commercial, or not-for-profit sectors.

## Data availability statement

No data associated with this submission.

## Ethical approval

Not applicable.

## Author contribution

Z.Y, M.J.T, and A.H conceived the idea, study concept and designed, A.H, M.J.T, Z.Y, M.S.A, H.S, and I.U. performed a literature review, data curation/interpretation and wrote the initial manuscript. Z.Y, M.S.A and I.U reviewed the manuscript and critically revised it to the final form. All authors approved the final version of the manuscript.

## Guarantor

Muhammad Sohaib Asghar and Zohaib Yousaf.

## Annals of medicine and surgery

None.

## Sources of funding

None.

## Consent

Not applicable.

## Registration of research studies

1. Name of the registry: N/A.
2. Unique Identifying number or registration ID: N/A.
3. Hyperlink to your specific registration (must be publicly accessible and will be checked): N/A.

## Declaration of competing interest

The authors declare no conflict of interest.
